# The Changing Shape of the Body Mass Index Distribution Curve in the Population: Implications for Public Health Policy to Reduce the Prevalence of Adult Obesity

**Published:** 2006-06-15

**Authors:** Alan D Penman, William D Johnson

**Affiliations:** Department of Medicine (Geriatrics), University of Mississippi Medical Center, Clinical Research Program; Department of Preventive Medicine, University of Mississippi Medical Center, Jackson, Miss

Many public health practitioners, policy makers, and epidemiologists worldwide have embraced Geoffrey Rose's population-based prevention concept, a strategy of disease prevention that aims to shift the population distribution of a risk factor in a favorable direction by applying interventions to an entire population (1-8). We believe that this may not necessarily be the best strategy for the prevention of adult obesity in populations and that the issue deserves closer examination and discussion. 

**Figure 1:**
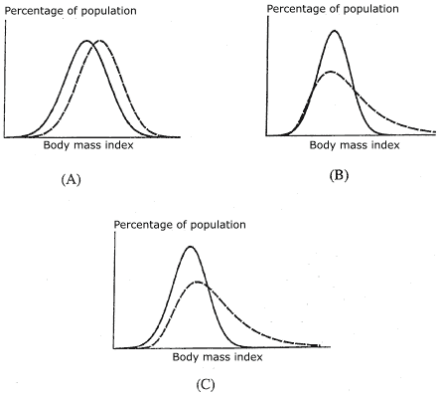
Possible changes over time in the population distribution of body mass index (BMI). Geoffrey Rose proposed that within a single population over time, an increase in the mean value of a risk factor and an increase in the corresponding prevalence of deviants would represent an upward shift, or a movement to the right (dashed curve) along the X-axis, in the entire population distribution of that risk factor (A) (7). We believe that the adult population distribution of BMI is more correctly described by a positively skewed distribution and that over time the degree of skewing has increased; that is, there is proportionately much more shifting of the distribution curve at the upper end than the lower (B and C).

Using cross-sectional data from INTERSALT (International Study of Sodium, Potassium, and Blood Pressure), a study of 52 centers in 32 countries with a range of geographic, social, and economic circumstances, Rose et al found strong, statistically significant correlations between the mean values of various cardiovascular disease risk factors and the corresponding prevalences of *deviants* (extreme values of that risk factor, at or above a certain cut point) (5). For example, there was a high, statistically significant correlation between the mean value of body mass index (BMI) and the prevalence of overweight; similar high correlations were found between the mean systolic blood pressure (BP) and the prevalence of hypertension, mean weekly alcohol intake and prevalence of heavy drinking, and mean urinary 24-hour sodium excretion and prevalence of high sodium intake (5-7). Comparison of the distributions of BP and BMI from the 32 countries showed large differences in the location of the curves on the X-axis (7). Based on these findings, Rose proposed that within a single population over time, an increase in the mean value of a risk factor and an increase in the corresponding prevalence of deviants would represent an upward shift, or a movement to the right along the X-axis, in the entire population distribution of the risk factor in question (Figure 1A). Referring to blood pressure, Rose stated: "Proportionately speaking, there is as much movement at the lower part of the blood pressure range as at the upper, with the 'coefficients of variation' (ratio of standard deviation to mean) being roughly constant" (7).

For public health policy, the implication of Rose's statement was that the reduction of disease prevalence requires the application of interventions to all members of the population, not just those in the "upper tail" of the distribution who are at greatest risk; the aim of this approach is to shift downward, or to the left along the X-axis, the entire population distribution of a risk factor. However, we believe there are several problems with this interpretation, at least with respect to the population distribution of BMI.

**Figure 2:**
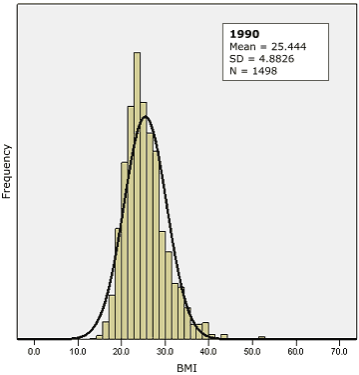
Population distribution of body mass index (BMI) with superimposed normal curve, Mississippi, 1990 (top) and 2003 (bottom). Cross-sectional data from the Mississippi adult population for 1990 through 2003 show that the population distribution of BMI is positively skewed and has become increasingly skewed over time. Source: Behavioral Risk Factor Surveillance System (13).

One problem is that Rose compared cross-sectional data from different countries, not from a single country at different times, and interpreted the results to mean that, in a single population over time, a change in the location of the distribution curve similar to the one he observed in data from more than one country would take place. In other words, a kind of evolution would occur in a population; for example, in response to changing dietary and lifestyle practices, the entire population distribution curve of a risk factor would move upward, or to the right (Figure 1A). However, Rose lacked longitudinal (or time series) data from a single population to support such an evolution of the distribution over time, stating merely that, "It is hard to see how it could fail also to apply to temporal changes within a population. . ." (5). The limited published data from same-population studies that show or discuss changes in the BMI distribution suggest that the population distribution of BMI has become increasingly skewed over time with little or no upward shifting of the entire distribution curve. For example, in the Minnesota Heart Health Program, the greatest increase over time in BMI for both men and women occurred in the upper part of the distribution curve (9). In an adult Norwegian population, the BMI distribution curve shifted to the right over time (10). A graph of National Health and Nutrition Examination Survey III data for 1988 through 1994 showed increasing skewness in the distribution of BMI for all sex–age groups and a greater shift in the upper part of the distribution (11,12). Finally, cross-sectional data from the Mississippi adult population for the years 1990 through 2003 show that the population distribution of BMI is positively skewed and has become increasingly skewed over time (Figure 2) (13).

**Figure:**
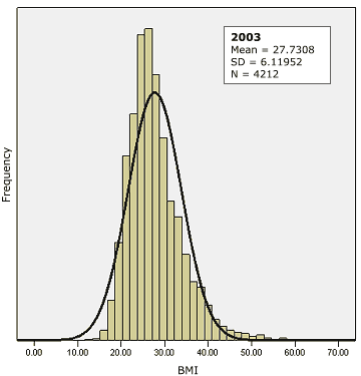

Data from cohort studies, in which data from the same individuals are collected over time, would help shed light on this issue by not only showing changes in the distribution of BMIs but also by allowing researchers to determine which individuals are gaining weight — all (or most) individuals in the cohort or predominantly those who already weigh more than average. Unfortunately, published data from large cohort studies on changes in BMI distribution are limited. One such study suggests that weight gain is greatest in those already overweight at baseline (14,15). An important limitation of such studies, however, is that the results may not be generalizable to the larger population.

Another problem is that the population distribution of BMI has been assumed, implicitly or explicitly, to follow a normal (Gaussian) distribution, or at least an approximately symmetric, bell-shaped distribution. However, this assumption has not been clearly proven or documented, and other distributions may provide a better fit. For example, from biologic considerations, a log-normal distribution of BMI might be expected. The values of many biologic variables are determined by multifactorial processes; if these processes have additive effects, then the values will be normally distributed (16-18). However, the growth of living tissues likely proceeds by multiplicative effects, and measures of body size (such as BMI) are more likely to follow a skewed, possibly log-normal, distribution (19-20). The log-normal distribution is always positively skewed, but the exact shape, or the degree of positive skewing, can vary depending on the parameters, and in certain situations it can resemble a normal Gaussian distribution (21). In Rose's published papers and monograph, the figures show positively skewed curves of varying degrees, most obviously for BMI (7). Asymmetrical, or skewed, distributions, such as the log-normal, can show a similar strong correlation between mean value and prevalence of deviants *without* a shifting of the entire curve. It is easy to demonstrate this by simulation: by progressively increasing the value of the coefficient of variation, a log-normal curve can be shown to change shape and become more positively skewed (lengthening the upper tail), with an increase in the mean and the prevalence of extreme values but with little shift in the lower end of the distribution. Empirical data also show this correlation between mean value and prevalence for skewed distributions. In the Mississippi adult population for the years 1990 through 2003, the correlation between mean BMI and prevalence of obesity was R = 0.93 (13).

We believe that the adult population distribution of BMI is more correctly described by a positively skewed distribution and that over time the degree of skewing has increased; that is, there is proportionately much more shifting of the distribution curve at the upper end than the lower (Figures 1B and 1C). The increasing skewing of the distribution of BMIs over time is consistent with physiologic studies of weight regulation and weight gain as well as clinical data, which show that the more a person weighs, the easier it is to gain more weight. In an obesogenic environment, the positive skewing of the distribution curve of BMIs increases over time as heavier individuals gain more weight than lighter individuals. Swinburn and Egger refer to this as the "the runaway weight gain train" (20).

These conclusions are based on limited national and state data, and further analysis is required. For example, BRFSS data from other states (which are available from 1990, when the survey began) could be examined. Mississippi may not be typical of the rest of the United States, but because it has the highest prevalence of obesity in the nation, it could be regarded as a bellwether state. Evaluating strategies for the prevention of adult obesity in populations is important because of the implications for public health policy — namely, we need to reconsider whether obesity prevention measures should be aimed primarily (but not, of course, exclusively) at those in the upper part of the distribution, particularly the upper tail. This refocusing suggests a return, in part, to the concept of *high-risk prevention*. Rose hinted at the possibility of this approach for weight reduction in the population (7), an approach that is especially important considering the J-shaped relationship between body weight (or BMI) and overall mortality (21).
